# Presurgical Optimization and Surgical Management of Extreme Adolescent Idiopathic Scoliosis: A Case Report

**DOI:** 10.7759/cureus.81987

**Published:** 2025-04-10

**Authors:** Eric Chun-Pu Chu

**Affiliations:** 1 Chiropractic and Physiotherapy Centre, New York Medical Group, Hong Kong, CHN

**Keywords:** adolescent idiopathic scoliosis (ais), chiropractic, chiropractor, scoliosis, scoliosis screening

## Abstract

We present the case of an 18-year-old Asian male patient with extreme adolescent idiopathic scoliosis (AIS) exceeding 180 degrees, who remained untreated for eight years following his initial diagnosis due to socioeconomic barriers and limited access to healthcare. At presentation, the patient measured 165 cm in height and weighed only 40 kg (BMI 14.7). He exhibited severe spinal deformity, compromised pulmonary function (forced vital capacity (FVC) at 65% of the predicted value), chronic pain (rated 5-7/10), and progressive functional decline that had led to withdrawal from traditional schooling. A multidisciplinary team implemented a presurgical optimization protocol incorporating halo-gravity traction, spinal manipulation, therapeutic exercises, respiratory rehabilitation, and nutritional intervention, all tailored through patient-specific 3D-printed spinal modeling. After eight weeks, the patient demonstrated remarkable improvements: a 2-cm height increase, 4-kg weight gain, improved pulmonary function (FVC 78% of the predicted value), and reduced pain reduction (2-3/10). These gains enabled successful posterior spinal fusion from T2 to L4. Postoperatively, pulmonary function normalized to 82% of the predicted value, and the patient continues with rehabilitation focused on functional restoration and pain management. This case uniquely contributes to the literature by documenting quantifiable improvements achieved through multidisciplinary presurgical optimization in extreme deformity. It demonstrates effective integration of multidisciplinary spine care, provides rare insight into the natural history of untreated severe AIS, and offers a comprehensive model for transitional care from conservative management to surgical intervention. The successful outcome, despite significant barriers, underscores the importance of adaptive, multidisciplinary strategies in managing complex spinal deformities, particularly in resource-limited settings.

## Introduction

Adolescent idiopathic scoliosis (AIS) is the most common spinal deformity affecting children and adolescents, with a prevalence of 2%-4% in the general population [1]. While the majority of cases involve moderate curvatures between 20 and 40 degrees, approximately 10% progress to severe deformities exceeding 45 degrees [1]. Curves exceeding 50 degrees are associated with higher progression rates into adulthood, often accompanied by accelerated structural deterioration and soft tissue degeneration, typically necessitating surgical intervention [2]. Extreme spinal curvatures exceeding 180 degrees represent the most complex and rare presentations of AIS. These cases are often associated with significant pulmonary compromise, nutritional deficiencies, and increased neurological risk. Current management guidelines recommend surgical correction as the standard of care for severe AIS [2]. However, patients with extreme deformities, impaired cardiopulmonary function, and poor nutritional status often face elevated surgical risks. This creates a paradox in which those most in need of corrective surgery may be deemed unfit for it. This dilemma is further amplified in resource-limited settings, where barriers to healthcare access can delay diagnosis and treatment until spinal deformities progress beyond conventional intervention thresholds.

Presurgical optimization for extreme scoliosis remains poorly documented in the literature, with limited consensus on effective multidisciplinary approaches [3]. Traditional presurgical protocols primarily emphasize halo-gravity traction as a standalone intervention, which has been shown to improve pulmonary function, reduce the severity of the deformity, and enhance the surgical tolerance of patients [4]. However, comprehensive approaches integrating respiratory rehabilitation, nutritional optimization, and complementary manual therapies are rarely reported. The role of conservative care providers in the management of complex spinal deformities has been largely underexplored. Recent technological innovations, such as patient-specific 3D-printed anatomical models, offer potential advantages for treatment planning in complex cases, but their use has largely been confined to surgical preparation rather than guiding conservative care. Furthermore, literature addressing extreme idiopathic curves exceeding 180 degrees in otherwise healthy adolescents is limited, creating significant knowledge gaps regarding optimal management strategies, potential for presurgical improvement, and expected outcomes following intervention.

This case report documents the successful multidisciplinary management of an 18-year-old male patient with extreme AIS exceeding 180 degrees, who presented after eight years without treatment. The case is notable for several unique features: the extraordinary magnitude of the idiopathic curve, the development and implementation of a comprehensive presurgical optimization protocol integrating halo-gravity traction with spinal manipulation and rehabilitation, the use of 3D-printed spine modeling to guide both conservative and surgical interventions, and the quantifiable improvements achieved despite the severity of the deformity. By detailing this integrated approach and its outcomes, we aim to contribute to the limited body of evidence on managing extreme spinal deformities, highlight the potential value of a multidisciplinary team including complementary medicine practitioners, and provide insights into presurgical optimization strategies for high-risk surgical candidates. This case helps bridge critical gaps in the literature and offers a potential model for managing similar complex cases, particularly in settings where treatment delays have allowed progression to extreme deformity.

## Case presentation

An 18-year-old Asian male patient with severe progressive AIS presented to the chiropractor with a history of spinal curvature initially identified at age 10 through a school screening program. Standing 165 cm tall and weighing only 40 kg (BMI 14.7), his severely underweight and emaciated appearance was directly attributable to his extreme spinal deformity. The patient is currently completing his high school education through a home-study program after his condition forced him to withdraw from traditional schooling at age 17. He lives with his parents and one sibling in a rural community approximately 80 km from the nearest specialized medical center.

The patient's medical history revealed no familial scoliosis or congenital spinal disorders across three generations, suggesting an idiopathic etiology. Born at term following an uncomplicated pregnancy and delivery, he achieved normal developmental milestones throughout early childhood. His parents first noticed subtle asymmetry in his shoulders and trunk around age 8, which progressed to a visible curve by age 10. Despite the early diagnosis, the patient received no medical intervention during his formative years due to a combination of financial constraints, limited healthcare access in his rural community, and parental fear of surgical procedures. The family attempted traditional remedies and massage therapies without success. His family history includes hypertension in his father and maternal grandmother but no connective tissue disorders or musculoskeletal conditions. The patient has never smoked, has no history of substance use, and reports no previous surgeries or drug allergies.

The kyphoscoliosis progressed steadily throughout adolescence, with marked acceleration during his growth spurt between ages 13 and 15. By age 14, he began experiencing intermittent episodes of low back pain, initially managed with over-the-counter acetaminophen. These episodes gradually increased in frequency and intensity, occurring 2-3 times weekly by age 16 and becoming nearly constant by age 17. The patient describes a dull, aching pain across the lower thoracic and upper lumbar regions (5-7/10 intensity), exacerbated by prolonged standing, physical activity, and cold weather. He occasionally experiences sharp, stabbing pain (8/10) with certain movements. Additional symptoms include increasing fatigue with minimal exertion, nocturnal discomfort affecting sleep quality, and progressive shortness of breath when climbing stairs or walking distances greater than 300 m.

**Figure 1:**
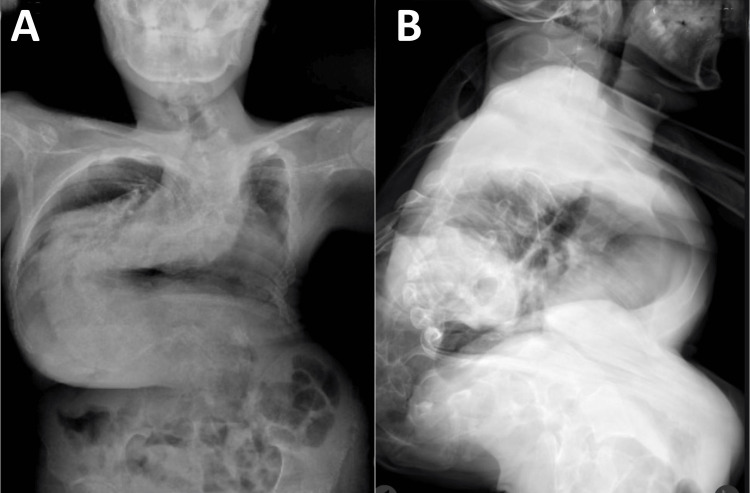
Presurgical X-ray of the spine: AP and lateral views (A) The anterior-posterior (AP) view demonstrates a severe right-sided thoracic scoliosis with a compensatory left-sided lumbar curve, forming a hook-like configuration from T1 to L5. (B) The lateral view reveals a marked loss of thoracic kyphosis and exaggerated lumbar lordosis, consistent with the compensatory mechanisms of severe scoliosis.

**Figure 2:**
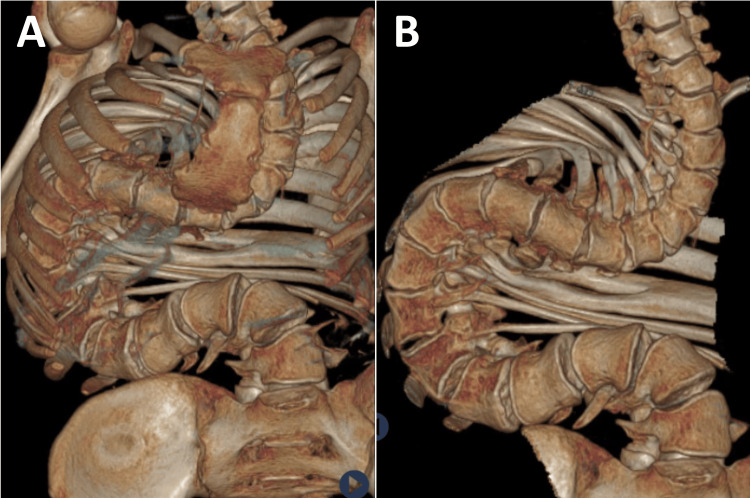
Full-spine computer tomography (CT) (A) The anterior CT reconstruction demonstrates a severe right-sided thoracic scoliosis with a compensatory left-sided lumbar curve, forming a hook-like configuration from T1 to L5. (B) The sagittal CT reconstruction reveals a marked loss of thoracic kyphosis and exaggerated lumbar lordosis, consistent with the compensatory mechanisms of severe scoliosis.

At age 17, an acute episode of severe low back pain (9/10) accompanied by increased respiratory difficulty prompted emergency care at the district hospital, representing his first formal medical evaluation since his initial diagnosis. Comprehensive testing included laboratory studies (complete blood count, metabolic panel, inflammatory markers, mineral levels), which returned normal results. Imaging studies revealed severe right thoracic and left lumbar scoliosis exceeding 180 degrees (Figure 1). CT scan confirmed the extreme deformity, showing significant vertebral rotation and thoracic cage deformation (Figure 2). Pulmonary function testing demonstrated a moderate restrictive pattern with forced vital capacity (FVC) at 65% of the predicted value. Echocardiography showed normal cardiac function with no pulmonary hypertension.

Despite the severity of his condition, the multidisciplinary team deemed the patient unsuitable for immediate surgical intervention due to the complexity posed by his extreme spinal deformity, poor nutritional status, compromised pulmonary function, and elevated risk of neurological complications. The hospital recommended nutritional supplementation, pulmonary rehabilitation, and pain management while seeking specialized opinions. During this process, he consulted with the chiropractor to explore conservative options for symptom control and possible curve stabilization. At the time, the patient was taking acetaminophen 500 mg PRN for pain, vitamin D3 1000 IU daily, calcium carbonate 600 mg twice daily, a daily multivitamin, and protein supplements twice daily.

On physical examination, the patient was able to ambulate independently, though with a mildly uneven gait. His posture demonstrated marked right thoracic prominence with compensatory left lumbar curve and significant trunk shift to the right of approximately 4 cm. Head tilt to the right was observed, with shoulder height asymmetry and pelvic obliquity. Vital signs showed a slightly elevated respiratory rate (20 breaths per minute) but normal blood pressure and heart rate. Neurological examination revealed intact sensory and motor functions throughout all extremities, with normal deep tendon reflexes and no evidence of pathological reflexes or clonus. The Adam's Forward Bend Test dramatically highlighted the rotational component of the deformity, with a prominent right thoracic rib hump measuring 4.5 cm using a scoliometer and a compensatory left lumbar prominence of 3.2 cm.

Spinal range of motion was significantly compromised in all planes, with thoracic motion most severely restricted. Muscular palpation revealed significant hypertonicity and spasm in the right thoracic paraspinal muscles, particularly the trapezius, rhomboids, and erector spinae, with compensatory tension in the left lumbar region. Chest expansion measured only 2 cm during deep inspiration, confirming the restrictive impact on pulmonary function. A review of imaging confirmed the extreme scoliotic curvature with severe vertebral rotation, significant rib cage deformity, and early degenerative changes at the curve extremities.

**Figure 3:**
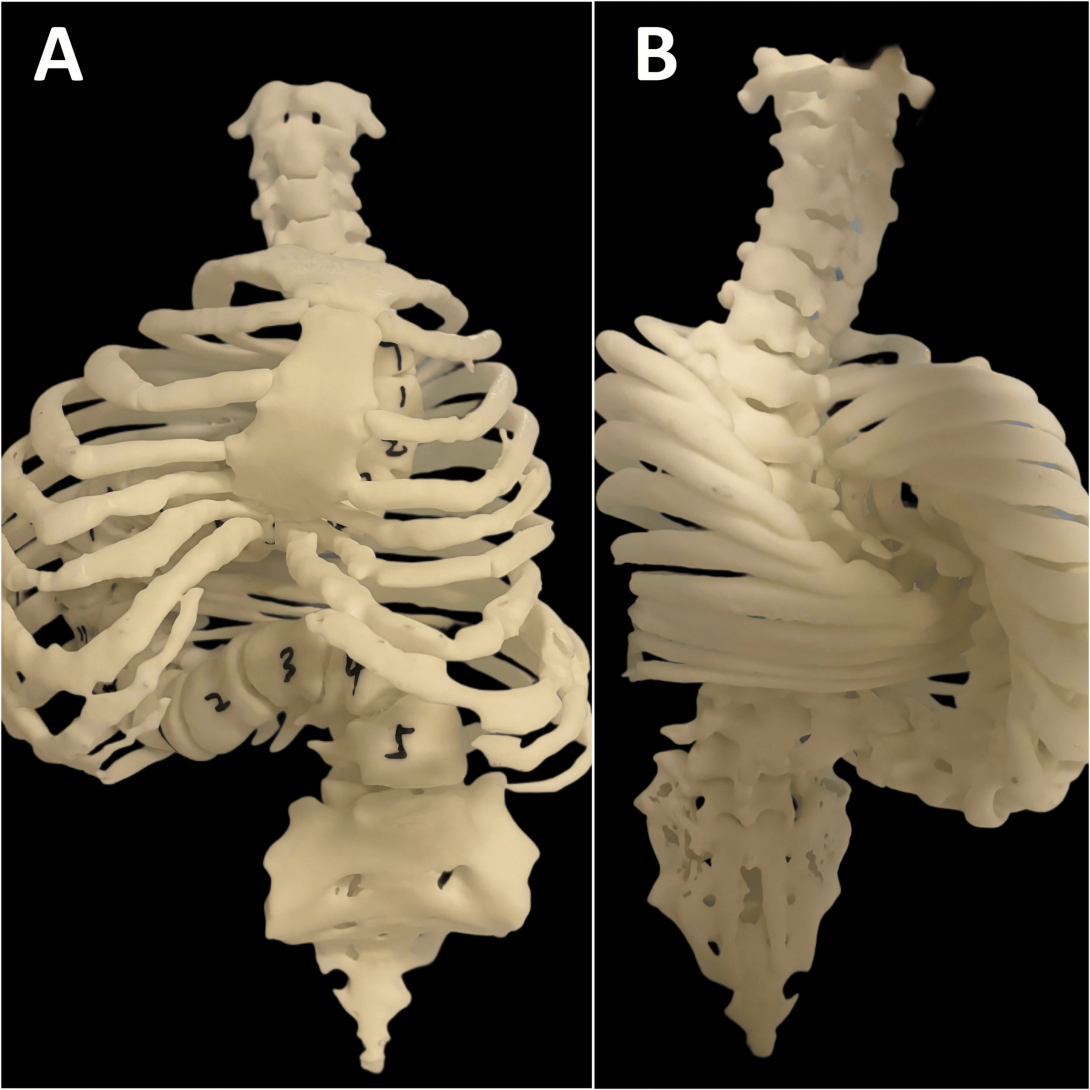
3D-printed spine model: anterior-posterior and posterior-anterior views (A) The 3D-printed spine model in the anterior-posterior (AP) view clearly demonstrates a severe right-sided thoracic scoliosis with a compensatory left-sided lumbar curve, forming a hook-like configuration from T1 to L5. (B) The posterior-anterior (PA) view of the 3D-printed model highlights the severe overlap of the ribs due to the extreme vertebral rotation and rib cage deformation.

The chiropractor collaborated with the medical team to develop an integrated presurgical optimization protocol rather than a definitive treatment, recognizing that the extreme curve would ultimately require surgical correction. A 3D-printed model based on CT imaging data was created to assist in treatment planning of spinal manipulation and surgery (Figure 3). The comprehensive regimen included daily halo-gravity traction beginning at 10% of body weight and gradually increasing to 40% over eight weeks; twice-weekly presurgical care featuring spinal manipulations, joint mobilization, and myofascial techniques; customized exercises; respiratory rehabilitation with balloon blowing and inspiratory muscle training; and nutritional intervention targeting weight gain. The patient demonstrated remarkable adherence to this protocol, attending all scheduled sessions and reporting 95% compliance with home exercises.

**Figure 4:**
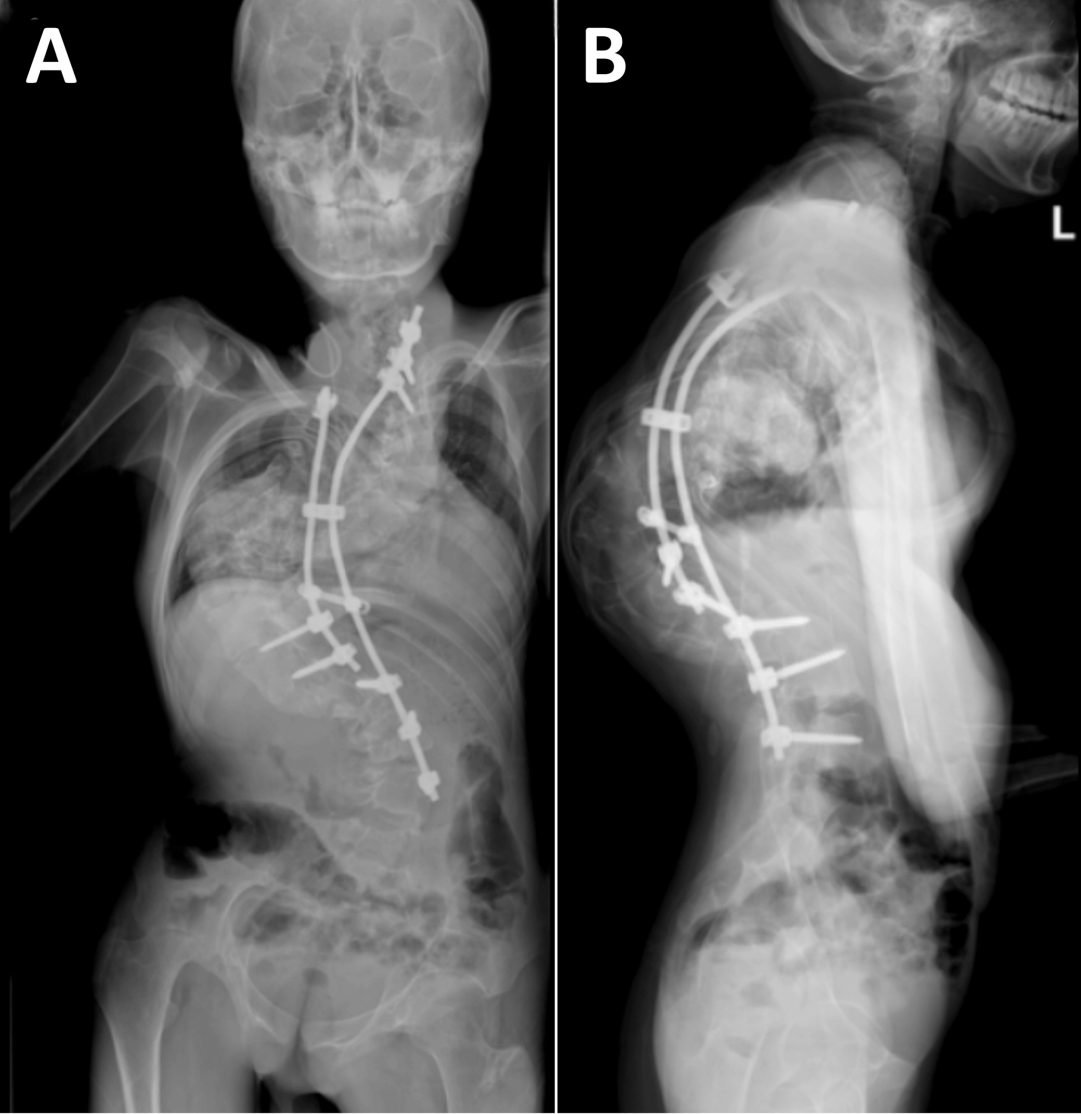
Postsurgical X-ray of the spine: AP and lateral views (A) The postsurgical anterior-posterior (AP) X-ray demonstrates residual scoliosis in the mid-thoracic region, though the severity of the deformity is notably reduced compared to preoperative imaging. (B) The postsurgical lateral X-ray reveals improved sagittal alignment, though some residual loss of thoracic kyphosis and increased lumbar lordosis remain.

After eight weeks of intensive multimodal therapy, the patient gained 2 cm in height (from 165 to 167 cm), increased weight by 4 kg, and demonstrated improved pulmonary function, with FVC rising from 65% to 78% of the predicted value. Pain reduced from baseline 5-7/10 to 2-3/10. Based on these improvements, particularly in nutritional status and pulmonary function, the surgical team deemed him suitable for staged surgical correction. The first stage of posterior spinal fusion with instrumentation from T2 to L4 was performed successfully, resulting in improvement in spinal alignment, though residual deformity remained due to the extreme nature of the initial curve (Figure 4).

Postoperatively, the patient's pulmonary function returned to normal range (FVC 82% of the predicted value), though he continued to experience intermittent neck and low back pain (3-4/10) after prolonged activities. After three weeks of inpatient rehabilitation, he was discharged home with a maintenance program focused on functional rehabilitation, pain management, respiratory exercises, ergonomic education, and psychological support. The patient continues to be monitored quarterly by the surgical team and receives monthly rehabilitation guidance. A second-stage surgery is being considered to address the cervical and upper thoracic alignment, but he refused. His current focus is on maintaining the surgical correction, continuing nutritional improvement, and gradually increasing functional capacity through an adapted exercise program.

## Discussion

This case of extreme AIS exceeding 180 degrees demonstrates several significant findings that contribute to our understanding of managing severe spinal deformities. The quantifiable improvements achieved through multidisciplinary presurgical optimization protocol, including a 2-cm height increase, 4-kg weight gain, 13% improvement in FVC, and pain reduction from 5-7/10 to 2-3/10, challenge traditional perspectives on the limitations of conservative care in very severe cases. These outcomes align with curve improvement, but the integrated approach produced additional functional and physiological benefits critical for surgical candidacy [5]. The successful presurgical optimization can significantly reduce perioperative risks in extreme deformity cases [6]. Notably, the comprehensive nature of our approach, combining traditional medical management with spinal manipulation [7], rehabilitative exercises [8], and respiratory training [9], addresses multiple physiological systems simultaneously, likely contributing to the positive outcome despite the severity of the deformity. The application of 3D-printed modeling for both manual therapy planning and surgical preparation represents an innovative extension of technology typically reserved for surgical contexts, allowing for precision in conservative care rarely documented in severe scoliosis cases.

Our case differs from previously published reports in several important aspects. Unlike the majority of extreme scoliosis cases in the literature, which typically involve neuromuscular, syndromic, or congenital etiologies, our patient presented with truly idiopathic scoliosis that progressed to an extraordinary magnitude due to socioeconomic and geographic barriers to healthcare access. This case provides rare documentation of the natural history of untreated AIS through adolescent growth in the modern era of early intervention. The multidisciplinary spine team represents a departure from traditional care models, which typically restrict complementary practitioners to managing milder musculoskeletal conditions. This collaborative approach bridges conventional divides between surgical and conservative paradigms, contrasting with siloed care models [10]. The effectiveness of our approach supports the current conceptual framework of integrative spine care, which proposes that complex spinal deformities benefit from diverse disciplinary perspectives throughout the care continuum [11,12]. Additionally, the detailed documentation of a transitional care pathway from initial conservative management through surgical intervention and postsurgical rehabilitation provides a comprehensive model rarely presented in case reports, which typically focus on either conservative or surgical management in isolation.

The implications of this case extend beyond its clinical success to raise important considerations for healthcare delivery, education, and research. The eight-year delay between diagnosis and treatment powerfully illustrates the consequences of healthcare disparities and argues for systemic improvements in screening and early intervention, particularly in underserved communities. Economically, while our multidisciplinary approach may seem resource-intensive initially, the prevention of catastrophic complications and improved surgical outcomes potentially reduce long-term healthcare costs, aligning with value-based care principles. From an educational perspective, this case demonstrates the importance of cross-disciplinary training and collaboration, suggesting that conventional medical, surgical, and complementary medicine curricula should incorporate more integrated content on complex spine management. Future research directions should include prospective studies comparing various presurgical optimization protocols, investigation of physiological adaptations enabling maintenance of function in extreme deformity, development of predictive models for identifying which severe cases might benefit most from intensive presurgical programs, and cost-effectiveness analyses of multidisciplinary approaches versus traditional care pathways. While a single case cannot establish definitive guidelines, this report provides a detailed template for managing similar complex cases and highlights critical knowledge gaps that merit further investigation, particularly as healthcare systems worldwide continue to encounter patients with advanced pathology due to treatment delays or limited access to specialized spine care.

## Conclusions

This case report demonstrates the successful management of an 18-year-old male patient with extreme AIS exceeding 180 degrees through an innovative multidisciplinary approach integrating traditional medical care, halo-gravity traction, manual therapy, and modern technologies such as 3D-printed anatomical modeling. The significant improvements in height, weight, pulmonary function, and pain achieved during the presurgical optimization phase, followed by successful surgical correction, highlight the potential benefits of comprehensive care models that transcend conventional disciplinary boundaries. This case contributes uniquely to the literature by documenting the natural history of untreated severe AIS, demonstrating the potential effectiveness of conservative presurgical optimization in extreme deformity, illustrating the valuable role complementary care providers can play within spine teams, and providing a template for transitional care from conservative management through surgical intervention. The socioeconomic and geographic barriers that delayed this patient's treatment for eight years underscore the critical importance of healthcare access, early intervention, and global health equity in preventing progression to such extreme deformities. As healthcare systems evolve toward more integrated and patient-centered models, this case serves as both a compelling example of successful collaboration across conventional and complementary disciplines and a call to action for improved early detection, intervention, and multidisciplinary management of spinal deformities worldwide. Future research should build upon this foundation to develop evidence-based protocols for optimizing surgical candidacy in high-risk cases, investigate the cost-effectiveness of integrated care approaches, and explore the physiological mechanisms that enable remarkable adaptation and recovery even in the most challenging spinal deformities.
